# Dietary intakes of hypertensive patients in rural India: Secondary outcomes of a randomised, double-blind, controlled trial

**DOI:** 10.1016/j.dialog.2023.100109

**Published:** 2023-02-03

**Authors:** Sudhir Raj Thout, Jie Yu, Joseph Alvin Santos, Md Hameed, Daisy H. Coyle

**Affiliations:** a The George Institute for Global Health India, Punjagutta, Hyderabad, India; b The George Institute for Global Health, University of New South Wales, Sydney, New South Wales, Australia; c Department of Cardiology, Peking University Third Hospital, Beijing, China

**Keywords:** Sodium, Salt, Hypertension, Salt substitute, India, Diet, Potassium

## Abstract

**Background:**

Hypertension is highly prevalent in India; however, little is known about the dietary intakes of those living with hypertension, particularly in rural areas. The primary aim was to assess the dietary intakes of individuals living in rural India with self-reported history of hypertension. As secondary analyses, we explored the dietary impact of a salt substitute in this population group.

**Materials and methods:**

This study used data from a large randomised controlled trial conducted in seven villages across rural India. Participants received either regular salt (100% sodium chloride) or the salt substitute (70% sodium chloride/30% potassium chloride) to replace all home salt use. Dietary intake at baseline and end-of-trial was assessed using 24-h dietary recalls. A range of dietary outcomes were assessed including energy intake, macronutrient intake and overall diet quality according to the Alternate Healthy Eating Index (AHEI).

**Results:**

A total of 454 participants were included in the analysis. At baseline, mean (SE) energy intakes in regular salt group and salt substitute group were similar at 5240 (110) kJ/day and 5120 (106) kJ/day, respectively. This was largely attributable to intakes of carbohydrates (74.4% of total energy intakes for regular salt group vs 75.4% for the salt substitute group) followed by total fat (15.8% vs 15.4%) and protein (10.4% vs 10.3%). Both groups also had similar AHEI scores at baseline, with mean (SE) total scores equating to 33.0 (0.4) (out of a total 90) for the regular salt group and 32.7 (0.4) for the salt substitute group. Both groups received lowest AHEI scores across the following components: vegetables, fruit and wholegrains. At baseline, the mean (SE) intakes of sodium across the regular salt and salt substitute groups were similar at 2349 (67) mg/day and 2396 (64) mg/day, respectively. In the salt substitute group, there was a significant reduction in total sodium intakes over time (−264 mg/day, 95% CI, −442 to −85), driven by the use of the salt substitute.

**Conclusion:**

This study found individuals with hypertension living in rural India had poor dietary intakes, including low intakes of fruits, vegetables and wholegrains, and high intakes of sodium. Salt substitutes may be an effective strategy for reducing sodium intake in this population group.

## 1 Introduction

Populations living in low and lower-middle-income countries are the most prone to diet-related diseases caused by inadequate energy intakes and micronutrient deficinies [1]. In India, there has been a significant national efforts since the 1950s to address micronutrient deficiency disorders [2], however, over the same period, health problems related to excess consumption of adverse nutrients have accelerated by ∼80% [3]. India is now experiencing a double burden of diet-related disease, characterised by nutritional deficiencies as well as the over-supply of adverse dietary components including energy, added sugar, sodium and unhealthy fats [4]. The rise in excess levels of adverse nutrients in India is partly related to the fast growing increase in the availability and heavy marketing of energy-dense, nutrient poor ultra-processed foods [5,6].

One of the biggest diet-related health concerns in India is the high prevalence of hypertension, which is a major modifiable risk factor for cardiovascular disease (CVD) and chronic kidney disease worldwide [[7], [8], [9]]. In India, an estimated 226 million (29.7%) adults are currently living with hypertension [[10], [11], [12]] and in 2019, more than 1.47 million deaths in India were attributable to hypertension [13,14]. Concerningly, only one-tenth of rural and one-fifth of urban individuals with hypertension achieve adequate blood pressure control [15], demonstrating the need for novel interventions to improve blood pressure control in India [16]. While there is a clear link between sodium and hypertension, few studies have explored overall dietary intakes in those with hypertension, particularly in rural India [16]. Such research would provide important insights into the dietary behaviour of this population group and ways in which diets could be modified to reduce rates of hypertension across this population.

Salt substitutes, which typically replace a portion of the sodium chloride in regular salt with sodium-free salts such as potassium chloride and magnesium chloride, have been recognised as a priority strategy to reduce population sodium intakes [17]. Such sodium-reduction strategies are crucial in places like India where the current average sodium intake for adults is ∼5 g/d of sodium (12.5 g/d of salt) [18,19]; far exceeding the World Health Organization's (WHO) maximum sodium intake recommendations of 2 g per day per person (5 g of salt) [20]. Salt substitutes are particularly effective for populations where a large portion of dietary sodium comes from salt added during cooking or at the table (discretionary salt use), such as in India [[21], [22], [23]]. In India, the contribution of discretionary salt to total sodium intakes is particularly high, especially for those living in rural areas where there is limited access to packaged foods and out-of-home foods e.g., restaurant and take-away foods, which are a major source of sodium in high-income countries [22,24].

Meta-analyses of randomised controlled trials (RCT) have demonstrated that salt substitutes are effective in reducing both systolic and diastolic blood pressure in hypertensive populations, without serious adverse effects [25,26]. Moreover, a recent cluster-randomised trial in China found that a five-year salt substitute intervention reduced the rate of stroke, major cardiovascular events and all-cause death among participants who had a hypertension and either a history of stroke or were 60 years of age or older [27].Meanwhile, in the Salt Substitute in India Study (SSiIS), the first salt substitute RCT conducted in rural India [28], participants who received the salt substitute intervention achieved a significant reduction in systolic and diastolic blood pressure and also had a significant increase in 24-h urinary potassium excretion and a decrease in the urinary sodium to potassium ratio [28].

While the SSiIS trial included an assessment of dietary intake pre and post intervention, these dietary outcomes were not assessed in the primary paper [28]. As there is currently very limited research exploring the diets of those living in rural India, particularly in those with hypertension, whereby diet is a key contributing factor to hypertensive status, the aim of this study was to conduct a secondary analysis of the SSiIS trial to examine the dietary intakes of individuals living in rural India with a self-reported history of hypertension. This study also explored changes in dietary intake over the course of the trial to assess whether the salt substitute intervention had an impact on diet quality.

## 2 Methods

### 2.1 Study design

The SSiIS was a three month, double-blind, RCT conducted in rural India between November 2019 and April 2020 [29]. Baseline assessments were conducted between November and December 2019, followed by intervention delivery in December 2019 to February 2020, and end-of-study assessments between March and April 2020. The study was approved by The George Institute for Global Health Ethics Committee (Project Number 09–2019). The study was registered in the clinicaltrials.gov database (NCT03909659) and was carried out according to the Declaration of Helsinki [30].

### 2.2 Participants

Eligible participants were recruited from seven villages in the Siddipet district of Telangana State in India. Villages were selected using purposive sampling based their proximity to the infrastructure required for this study and participants were recruited by study staff who door knocked on potential households. To be eligible, individuals were required to be aged 18 years or over, have a self-reported history of hypertension diagnosed by a health professional and reported that they prepare and consume most of their meals at home. Due to concerns over potassium-enriched salt-substitutes and the risk of hyperkalemia [31], participants were excluded if they or any of their household members take potassium-sparing diuretics, potassium supplements (but not if they used other hypertensive medications such as Angiotensin-converting enzyme inhibitors or Angiotensin receptor blockers), or have any known acute or chronic kidney disease [32]. Individuals were also excluded if they (or any of their household members) had concerns regarding the use of salt substitutes, were not expected to live longer than six months from the date of assessment or had other household members already enrolled in the trial. A study physician was involved during the recruitment stage to check the validity of the self-reported hypertension status and to confirm the presence or absence of serious kidney disease.

As described previously [28], participants were randomly assigned to the intervention and control groups in a 1:1 ratio through a central computerized process. The study team were blinded to the randomisation sequence and were unaware of randomisation assignment [29]. Due to the nature of the intervention whereby all household members are potentially exposed to the intervention, written informed consent was obtained from the main study participant as well as all household members. For household members under 18 years of age, informed consent was provided by a parent or guardian.

### 2.3 Intervention and control

Participants in the intervention group received a reduced sodium, added potassium salt substitute made up of 70% sodium chloride and 30% potassium chloride. Whereas those in the control group received regular salt (100% sodium chloride). Both salts were fortified with iodine as per the Indian regulatory requirements [28]. All participants were advised to replace all discretionary salt used in the household during food preparation, cooking and at the table with the study salt provided and were directed to continue to use the study salt until the end of the three-month study period. Households were provided with 20 g/person/day of the study salt to ensure it would be sufficient in covering the full amount of discretionary salt use per household. The salt substitute was made by Siddharth Starch Pvt. Ltd., a company based in Maharashtra, India, who were commissioned to blend and supply the product. Both salts were provided free of charge and were provided in identical plain packaging. The study salts were delivered directly to the homes of the participants by the field researchers.

### 2.4 Data collection

The methods for data collection and measurements have been published elsewhere [28]. In brief, trained field researchers conducted five face-to-face visits at the homes of participants over the course of the study. During these visits, researchers collected blood pressure measurements, 24-h urine samples and information about the acceptance and usage of the study salt. Dietary intake was also assessed using paper-based, multi-pass 24-h dietary recalls. One 24-h dietary recall was performed at each time point - baseline and end of the trial. The dietary recalls were administered by 10 trained field researchers with training and experience in the collection of multi-pass 24-h dietary recalls. To assist with reporting of portion size estimates, participants were provided with a food model booklet containing photographs of different sized food and beverage containers such as bowls, shapes, mounds and drinking glasses and were asked to estimate intake according to these standard sizes. For mixed dishes, participants were asked to provided information about individual ingredients and quantities for the whole dish along with a brief description of the cooking method. Discretionary salt included the amount of salt added to food during cooking and at the table. Data was collected only for the recruited participant (not all household members).

### 2.5 Nutrition composition data

Nutrition data collected from 24-h dietary recall surveys were transcribed into a purpose-built nutrient database. Foods were categorised into 12 major food groups (e.g., beverage: non-alcoholic) and 23 minor food groups (e.g., wine, coffee, tea). The nutrition composition for each item was obtained from the Indian Food Composition Tables (2016), developed by the Indian National Institute of Nutrition (2016) [33], and was supplemented with nutritional composition data collected from the labels of all packaged food products sold at Indian supermarkets in Delhi and Hyderabad between 2012 and 2014 [34].

### 2.6 Diet quality assessment

The quality of participants' diets was assessed using the Alternate Healthy Eating Index (AHEI-2010) [35]. The AHEI-2010 is a measure of diet quality that incorporates 11 food and nutrient components that strongly predict major chronic disease risk [35,36], CVD [37,38] and cancer [[37], [38], [39]]. These components include vegetables, fruit, wholegrains, sugar-sweetened beverages and fruit juice, nuts and legumes, red/processed meat, trans fat, long-chain (n-3) fats, polyunsaturated fatty acids (PUFA), sodium and alcohol [35]. Each component is given a score between 0 and 10 depending on intake. A higher score indicates higher diet quality and the maximum dietary score is 110 [35].

Due to a lack of nutrition data pertaining to each component of the AHEI-2010, the current study used a modified AHEI-2010 that included nine of the 11 components. To calculate the dietary score for each component, we first calculated total daily intake (in serves/day, or mg/day for sodium, or drinks/day for alcohol). This was then converted into a score (between 0 and 10) based on the AHEI-2010 weighting scheme [35]. The scoring system for each component is described in Supplementary Table 1.

### 2.7 Statistical analysis

The planned sample size for the trial was 498 participants to provide 80% power with a significance level of 5% (1-sided test) to detect a 5 mmHg or greater difference in mean systolic blood pressure between groups [29]. Continuous data were shown as means with SD (or SE). Categorical data were presented as numbers with corresponding percentages. Participants with missing 24-h dietary recall data at either baseline or follow-up were excluded from the analysis. The within and between group changes in diet (energy, macronutrient, AHEI-2010 scores, sodium, and potassium) over the course of the trial (baseline to 3 months) were assessed by linear mixed effects model that adjusted for baseline measurements and the repeated measures within subject.

The percent sodium and potassium contribution of each food category and subcategory were then calculated for each participant by dividing the amount of sodium and potassium consumed from each food category by the total amount of sodium and potassium consumed across the entire diet x 100. The mean percent contributions were then calculated and compared between the salt substitute and regular salt group. Two-sided *p* < 0.05 was considered statistically significant. All statistical analyses were conducted using STATA 15.1 (StataCorp LP, College Station, TX, USA) and SAS version 9.2, SAS Enterprise Guide version 7.1.

## 3 Results

In total, 502 participants met the inclusion criteria and provided consent to be included in the SSiIS trial. A total of 48 participants were excluded from the current study due to missing 24-h dietary recall data at either baseline or follow-up (Supplementary Fig. 1). Of the remaining 454 participants, 217 (47.8%) were from the regular salt group (control) and 237 (52.2%) were from the salt substitute group (intervention) (Supplementary Fig. 1). Baseline characteristics of all included participants are presented in Table 1. There were no appreciable differences in baseline characteristics between participants in both groups (Table 1).

**Table 1:** Baseline characteristics of participants with dietary recall data in the Salt Substitute in India Study.

	Regular salt (*n* = 217)	Salt substitute (*n* = 237)	Total (*n* = 454)	*P* value for mean difference between groups
Age, years, mean (SD)	61.6 (12.8)	61.5 (11.1)	61.6 (11.9)	0.93
Sex, F, no. (%)	130 (59.9)	140 (59.1)	270 (59.5)	0.86
Education, no. (%)				0.70
Primary school or lower	187 (86.2)	206 (86.9)	393 (86.6)	
High school^1^	25 (11.5)	28 (11.8)	53 (11.7)	
College or higher	5 (2.3)	3 (1.3)	8 (1.8)	
Current Smoker, no. (%)	10 (4.6)	15 (6.3)	25 (5.5)	0.42
Past smoker, no. (%)	16 (7.4)	20 (8.4)	36 (7.9)	0.67
Alcohol consumption, current^2^, no. (%)	30 (13.8)	41 (17.3)	71 (15.6)	0.31
Alcohol consumption, past^2^, no. (%)	61 (28.1)	68 (28.7)	129 (28.4)	0.89
Medication use, no. (%)				
Antihypertensive agents	206 (94.9)	230 (97.1)	436 (96.0)	0.25
Diuretic	1 (0.5)	0 (0)	1 (0.2)	0.48^4^
Calcium channel blockers	7 (3.2)	6 (2.5)	13 (2.9)	0.78^4^
ACEI or ARB	68 (31.3)	65 (27.4)	133 (29.3)	0.36
Beta-blocker Other	44 (20.3)	58 (24.5)	102 (22.5)	0.28
Alpha-blockers	88 (40.6)	102 (43.0)	190 (41.9)	0.59
Statin or other lipid lowering drugs	1 (0.5)	1 (0.4)	2 (0.4)	1.00^4^
BMI, kg/m^2^, mean (SD)	23.6 (4.1)	23.2 (4.7)	23.4 (4.4)	0.37
SBP, mmHg, mean (SD)	131 (22)	133 (20)	132 (21)	0.34
DBP, mmHg, mean (SD)	83 (13)	84 (12)	83 (12)	0.32
History of diabetes, no. (%)	41 (18.9)	59 (24.9)	100 (22.0)	0.12
24-h urine electrolytes level^3^, mean (SD)				
Sodium, milligrams	3620 (1640)	3800 (1870)	3720 (1770)	0.28
Potassium, milligrams	840 (460)	820 (440)	830 (450)	0.74

SD: standard deviation; CVD: cardiovascular disease; SBP: systolic blood pressure; DBP: diastolic blood pressure; ACEI: angiotensin-converting enzyme inhibitors; ARB: angiotensin receptor blockers.

1 High school includes junior high school, senior high school and technical secondary school.

2 Defined as currently consuming any type of alcohol.

3 After excluding samples that were likely incomplete, the total number of urine samples eligible for analyses was 418, including 221 in the salt substitute group and 196 in the regular salt group.

4 Fisher's Exact Test.

### 3.1 Dietary intake and diet quality of study population at baseline

Mean (SE) energy intakes in regular salt group and salt substitute group at baseline were similar at 5240 (110) kJ/day (i.e., 1252 (26) Kcal/day) and 5120 (106) kJ/day (1224 (25) Kcal/day), respectively (Table 2). This was largely attributable to intakes of carbohydrates which made up 74.4% of total energy intakes for regular salt group vs 75.4% for the salt substitute group. This was followed by total fat (15.8% vs 15.0%) and protein (10.4% vs 10.3%). Intakes of PUFA were also similar across groups at 3407 (165) mg/day vs 3247 (158) mg/day, respectively (Table 2). Both groups also had similar AHEI scores at baseline, with mean (SE) total scores equating to 33.0 (0.4) (out of a total 90) for the regular salt group and 32.7 (0.4) for the salt substitute group. Both groups received lowest scores across the following components: vegetables, fruit and wholegrains (Supplementary Table 2).

**Table 2:** Change in energy, macronutrient and diet quality scores.

	Regular salt (n = 217)			Salt substitute (*n* = 237)			Mean diff between groups at end of trial (95% CI)	*P*-value for mean diff^1^
	Baseline	End of trial	Mean change within group (95% CI)	Baseline	End of trial	Mean change within group (95% CI)		
Energy (kJ/day)	5240 (110)	5696 (110)	456 (150 to 761)	5120 (106)	6201 (106)	1081 (789 to 1373)	626 (203 to 1048)	0.004
Protein								
g/day	33.5 (1.1)	34.1 (1.1)	0.5 (−2.4 to 3.5)	32.4 (1.0)	37.3 (1.0)	4.9 (2.1 to 7.8)	4.4 (0.3 to 8.5)	0.037
% energy	10.4 (0.2)	9.7 (0.2)	−0.7 (−1.1 to −0.2)	10.3 (0.2)	9.8 (0.2)	−0.4 (−0.9 to 0.0)	0.3 (−0.4 to 0.9)	0.444
Carbohydrate								
g/day	232.7 (4.7)	254.4 (4.7)	21.7 (8.7 to 34.6)	229.4 (4.5)	266.3 (4.5)	36.9 (24.5 to 49.3)	15.2 (−2.7 to 33.1)	0.097
% energy	74.4 (0.4)	75.5 (0.4)	1.1 (−0.1 to 2.3)	75.4 (0.4)	72.0 (0.4)	−3.4 (−4.6 to −2.3)	−4.5 (−6.2 to −2.8)	<0.001
Total fat								
g/day	22.3 (1.0)	24.7 (1.0)	2.4 (−0.3 to 5.1)	21.0 (0.9)	31.8 (0.9)	10.8 (8.2 to 13.3)	8.4 (4.7 to 12.1)	<0.001
% energy	15.8 (0.4)	15.4 (0.4)	−0.4 (−1.4 to 0.6)	15.0 (0.4)	18.9 (0.4)	4.0 (3.0 to 5.0)	4.4 (2.9 to 5.8)	<0.001
PUFA^2^								
mg/day	3407 (165)	3864 (165)	456 (−2 to 914)	3247 (158)	4851 (158)	1604 (1166 to 2043)	1148 (514 to 1782)	<0.001
% energy	2.4 (0.1)	2.4 (0.1)	0.0 (−0.2 to 0.2)	2.3 (0.1)	2.9 (0.1)	0.6 (0.4 to 0.8)	0.6 (0.3 to 0.8)	<0.001
Total AHEI score^3^	33.0 (0.4)	31.9 (0.4)	−1.1 (−2.2 to 0.0)	32.7 (0.4)	32.3 (0.4)	−0.4 (−1.5 to 0.6)	0.7 (−0.9 to 2.2)	0.402

Results presented as mean (SE).

1 Linear mixed model; adjusted for baseline measurements and accounted for repeated measurements within subject.

2 Polyunsaturated fatty acids, PUFA.

3 AHEI score ranges from 0 (lowest) to 90 (highest).

### 3.2 Main dietary sources of sodium and potassium at baseline

At baseline, the mean (SE) intakes of sodium across the regular salt and salt substitute groups were similar at 2349 (67) mg/day and 2396 (64) mg/day, respectively. Across both groups, the majority of sodium intake was attributable to discretionary salt use, which accounted for 90–91% of total sodium intakes (Table 3). Besides discretionary salt, the major sources of sodium across both groups included fruits and vegetables (contributing to 4–5% of total sodium intakes), dairy and dairy products (2–3%), cereal, grains and products (1%), meat, poultry and eggs (1%), and condiments (0–1%) (Supplementary Table 3).

**Table 3:** Change in sodium consumption between groups over course of the trial.

	Regular salt (n = 217)			Salt substitute (n = 237)			Mean diff between groups at end of trial (95% CI)	P-value for mean diff^1^
	Baseline	End of trial	Mean change within group (95% CI)	Baseline	End of trial	Mean change within group (95% CI)		
Percent contribution of discretionary salt, mean (SE)	91 (0)	90 (0)	−1 (−2 to 0)	90 (0)	90 (0)	0 (−2 to 1)	1 (−1 to 2)	0.525
Sodium consumption (from discretionary salt), mg/day, mean (SE)	2166 (65)	2144 (65)	−22 (−203 to 160)	2202 (63)	1936 (63)	−265 (−439 to −92)	−244 (−494 to 7)	0.057
Sodium consumption (from all foods), mg/day, mean (SE)	2349 (67)	2335 (67)	−14 (−200 to 173)	2396 (64)	2132 (64)	−264 (−442 to −85)	−250 (−508 to 8)	0.057

1 Linear mixed model; adjusted for baseline measurements and accounted for repeated measurements within subject.

The mean (SE) potassium intake at baseline was 1173 (38) mg/day for the regular salt group and 1136 (36) mg/day for the salt substitute group, of which discretionary salt contributed to 1% of total intakes (Table 4). The major food group sources of potassium at baseline across both groups included fruits and vegetables (contributing to 53–57% of potassium intakes), cereal, grains and products (25–26%), dairy and dairy products (12–14%) and meat, poultry and eggs (5%) (Supplementary Table 4).

**Table 4:** Change in potassium percent contribution/consumption.

	Regular salt (n = 217)			Salt substitute (n = 237)			Mean diff between groups at end of trial (95% CI)	P-value for mean diff^1^
	Baseline	End of trial	Mean change within group (95% CI)	Baseline	End of trial	Mean change within group (95% CI)		
Percent contribution of discretionary salt, mean (SE)	1 (0)	0 (0)	0 (0 to 0)	1 (0)	1 (0)	0 (0 to 0)	0 (0 to 1)	0.064
Potassium consumption (from discretionary salt), mg/day, mean (SE)	7 (4)	6 (4)	0 (−10 to 10)	10 (3)	12 (3)	2 (−7 to 12)	3 (−11 to 16)	0.716
Potassium consumption (from all foods), mg/day, mean (SE)	1173 (38)	1226 (38)	53 (−51 to 157)	1136 (36)	1310 (36)	173 (74 to 273)	121 (−23 to 265)	0.101

1 Linear mixed model; adjusted for baseline measurements and accounted for repeated measurements within subjects.

### 3.3 Effects of the salt substitute intervention on dietary intake

Over the course of the trial, there was a significant increase in the total daily consumption of energy and carbohydrates for both groups (Table 2). Moreover, compared to the regular salt group, the salt substitute group had a significantly greater increase in daily energy and protein intake, by 626 kJ/day (95% CI, 203 to 1048 kJ/day) and 4.4 g/d (0.3 to 8.5), respectively. Similarly, the salt substitute group also had a significantly greater increase in both total fat and polyunsaturated fatty acids (PUFA) intake compared to the regular salt group at 8.4 g/d (4.7 to 12.1) and 1148 mg/day (514 to 1782), respectively (Table 2). There was no difference in total AHEI scores between groups at baseline and there was no difference between groups over time.

### 3.4 Effects of the salt substitute intervention on sodium and potassium intakes

Over the course of the trial, there were no significant differences between the regular salt and salt substitute group in terms of reductions in total sodium intake (mg/day) or percent contribution of discretionary salt to total sodium consumption (Table 3). However, within the salt substitute group, there was a significant reduction in total sodium intakes by −264 mg/day (95% CI, −442 to −85 mg/day), which was not observed in the regular salt group. This reduction was primarily attributable to reductions in sodium from discretionary salt use (Table 3). Similar to sodium, there were no significant differences between the regular salt and salt substitute group in terms of the change in percent contribution and consumption (mg/day) of potassium (Table 4). However, within the salt substitute group, there was a significant increase in total potassium intakes by 173 mg/day (95% CI, 74 to 273 mg/day), which was not observed for the regular salt group. Considering changes in discretionary salt use contributed to a 2 mg reduction in potassium intake, the changes in potassium consumption was primarily driven by changes to diet.

## 4 Discussion

This secondary analysis of a salt substitute RCT assessed the dietary intakes of hypertensive individuals living in rural India. Baseline data demonstrated that this population group had poor dietary intakes, including a low intake of fruits, vegetables, wholegrains, protein and potassium and a high intake of sodium. Over the three-month trial, consumption of energy and carbohydrates increased for both groups, which was surprising given the trial only targeted discretionary salt use. Within the salt substitute group there was a significant reduction in sodium intakes and a significant increase in potassium intake; changes that were not observed in the regular salt group. Overall, our data suggest that there is a strong need to improve dietary intakes of hypertensive patients in rural India and that salt substitutes may be an effective way to improve diets, particularly with regard to sodium consumption.

Dietary quality is recognised as a key factor influencing the nutritional status of populations worldwide [40,41]. A healthy diet is also key for optimal nutrition and health outcomes through all stages of life, with poor diet a major risk factor for the development of non-communicable diseases (NCDs), leading to early onset morbidity and mortality [42]. Despite this, there has been limited research to-date exploring the quality of diets in India. An analysis of the 2011–12 National Nutritional Monitoring Bureau data identified low average vegetable consumption among men at 143 g/person/day for men and 138 g/person/day for women [43]. Moreover, a recent analysis of the nationally representative National Sample Survey (2011−2012) indicated that per capita household consumption of fruit and vegetables was 160 g/day in rural India and 184 g/day in urban India [44]; far short of the WHO benchmark recommendation of 400 g/person/day [45]. Our findings are consistent with this prior literature, with our paper demonstrating that this population has very poor dietary intake, including low intakes of fruit and vegetables and well as low intakes of wholegrains, and nuts and legumes. These foods have been shown to be beneficial for the management of hypertension as they contain a number of health-protective properties including high levels of potassium, fibre, magnesium and healthy fats such as Omega 3 s [[46], [47], [48]].

Our study also identified high levels of sodium consumption in this population group. Using 24-h diet recall data, it was estimated that this population consumes approximately 2500 mg of sodium each day (∼6.25 g of salt). This is likely an underestimate compared to urinary sodium, given the methodological limitations associated with 24-h diet recalls [49] as well as the fact that the 24-h urinary sodium estimates collected as part of the primary study estimated that average sodium intakes in this population group were ∼ 3700 mg/d [28]. Nonetheless, it is clear that sodium intakes in this population are a huge concern, with average intakes far exceeding the WHO recommended limit of 2000 mg per person each day [50]. Importantly, most of this intake (>90% of total sodium intakes) was attributable to discretionary salt use. This is consistent with prior research, including a cross-sectional survey of 1283 Indian participants, which found that discretionary salt accounted for 87.7% of sodium intakes in South India and 83.5% in North India [22]. These findings support the need for effective strategies to reduce sodium consumption in rural India, particularly from discretionary salt. Salt substitutes could play in important role in achieving these reductions and to help reduce the alarmingly high rates of hypertension [25,42].

Another key finding from this study is that mean potassium intakes were around 1.2 g per day per person, far below the WHO recommendations of 3.5 g per day for adults [10]. It has been widely acknowledged that a potassium-rich diet favours blood pressure reduction [51] as well as extenuate the impact of high salt intake on blood pressure [52,53]. In this study, mean potassium consumption from discretionary salt was relatively low, which is likely driven by the fact that the salt substitute was a 70% sodium chloride and 30% potassium chloride blend. This suggests that greater benefits to potassium intake may be able to be achieved through increasing the proportion of potassium in salt substitute products. Moreover, changes to diet, particularly greater intake of fruits and vegetables would also play a key role in increasing potassium intake in this population group. Nonetheless, inadequate potassium intake in this study population highlights that actions to increase dietary potassium particularly among people with high blood pressure in rural India are needed.

There were no detectable differences in changes to sodium and potassium consumption across the two groups. However, we did find that sodium intakes did reduce over the course of the study for the salt substitute group, which is consistent with 24-h urinary results from the primary study [28]. This finding has important policy implications. As the primary outcomes from the SSiIS study [28] found that no participants had heard of reduced‑sodium salt at baseline, our data suggests that public health promotion and increased availability of salt substitutes for hypertensive patients could be an effective and cost-effective strategy to reduce sodium intakes within this population group [28]. Surprisingly, potassium consumption also increased significantly in the salt substitute group, and this was driven by food (not the salt substitute). These findings suggest that the salt substitute intervention may have had an unexpected impact on the diets of participants. As nearly all participants considered high salt intake to be bad for health and reported trying to eat less salty foods [28], perhaps participation in this study could have indirectly influenced dietary intakes as seen in other behaviour change interventions [54]. It is also possible that given this was a three-month long study, changes in availability of fruits and vegetables due to seasonality could have contributed to some of the changes in potassium intakes seen. Future research examining assessing the impact of salt-substitutes in other populations is required to explore these findings further.

Unhealthy diet is one of the modifiable risk factors in the prevention of noncommunicable diseases [42,55]. Consistent with prior research, we found that diets were predominately plant-based, making up approximately three quarters of total energy intakes in our population group [56]. On the contrary, protein intakes were relatively low, making up approximately 10% of total energy intakes. This is in line with prior research conducted in India, which found that individuals had low protein intakes and that protein intakes have reduced protein over time [56]. These results are somewhat expected given a large proportion of the Indian population is vegetarian [57,58], and that we observed very low intakes of red meat in the current study. Nonetheless, the low energy and protein intakes observed in this study are a concern given they do not meet recommended levels [59,60]. This may be partly explained by the 24-h recall method used to estimate dietary intake; we only collected one recall at each time point (at the same time as the 24-h urine samples), which may not be representative of usual intake and may have underestimated energy and nutrient consumption [61,62]. Nonetheless, self-report dietary data provides good insight into food preferences and patterns of eating that may be missed by biomarkers alone [63].

This study has several key strengths. This includes a randomised controlled design, the use of blinding and the high completion rate of participants with dietary intake data. We assessed dietary intakes across macronutrients, sodium, and potassium, and we also explored dietary quality of participants using the AHEI score, allowing for a comprehensive assessment of dietary intake. Assessing dietary intakes pre and post study also allowed us to assess changes in diet in response to the salt-substitute intervention, providing novel insights into the dietary impact of salt-substitutes in hypertensive populations in India.

Some limitations need to be noted. As only one 24-h dietary recall was collected at each time point, this data may not reflect habitual intakes and may have underestimated actual intakes [61,62]. We acknowledge that collecting additional dietary intake data through other dietary intake assessment methods, such as food frequency questionnaires, may have resulted in more accurate estimates of dietary intake. However, it was not possible to conduct these additional dietary intake methods due to time and financial constraints. As the RCT was restricted to seven villages in one state in India due to logistical and budgetary constraints, our findings may not be generalisable to other Indian hypertensive populations, such as those living in urban areas with different dietary sources of sodium. As such, there is a need for future research that uses gold-standard, rigorous dietary assessment methods to explore the dietary intakes of those living in rural India, particularly those with hypertension, to confirm whether intakes of protein and energy are consistent with the findings observed in this study. Moreover, this research has identified that discretionary salt use remains a substantial contributor to sodium intakes in rural India, highlighting the potential of salt-substitutes as a method for reducing sodium consumption. Moreover, given results from the SSaSS trial found that salt substitutes are a cost-saving intervention for the prevention of stroke in low-middle income countires [11], further consideration of salt substitutes by national-level policymakers in such countries is warranted. Lastly, future studies are needed to understand the benefit-risk profile of potassium-based salt substitutes at the population level, including among those with pre-existing co-morbidities such as chronic kidney disease [28].

## 5 Conclusion

In conclusion, this study demonstrated that hypertensive patients in rural India have poor dietary intakes, particularly low intakes of fruits, vegetables, wholegrains, protein and potassium. Sodium consumption in this population was high, and this was largely attributable to discretionary salt use. The study also found that replacement of regular salt with a reduced‑sodium added‑potassium salt for home use for three months led to substantial reductions in sodium intake and therefore may be a low-cost and effective strategy for reducing sodium intake in this population group.

## Contributors

SRT conceived and designed the study, contributed to data collection, developed nutrition composition data and assisted in drafting the manuscript. JY conceived and designed the study, contributed to data analysis and assisted in drafting the manuscript. JAS developed the analysis plan, analysed and interpreted the data, and participated in preparation of the manuscript. MH contributed to data collection and development of nutrition composition data and participated in preparation of the manuscript. DHC contributed to study design, interpreted the data, assisted in drafting the manuscript and supervised all aspects of the study. All authors had full access to all the data in the study and accept responsibility to submit for publication. SRT and JAS directly accessed and verified the underlying data reported in the manuscript.

## Funding

The study was funded by the George Institute for Global Health Seed Grant (Grant number 0141030). The funders had no role in the design of the study, in the collection, analyses, or interpretation of data, in the writing of the manuscript, or in the decision to publish the results.

## Declaration of Competing Interest

All other authors declare no competing interests.

## Data Availability

De-identified data that support the findings of this study are available from the corresponding authors upon reasonable request following the publication of this article.

## Appendix A Supplementary data

Supplementary material 1. Participant flow diagram Image 1

Supplementary material 2. Criteria for minimum and maximum AHEI scores Image 2
