# Supplementary figures and images for: Dietary intakes of hypertensive patients in rural India: Secondary outcomes of a randomised, double-blind, controlled trial
Source: Dialogues Health. 2023 Feb 3;2:100109. doi: 10.1016/j.dialog.2023.100109 (PMC10953904; doi:10.1016/j.dialog.2023.100109)

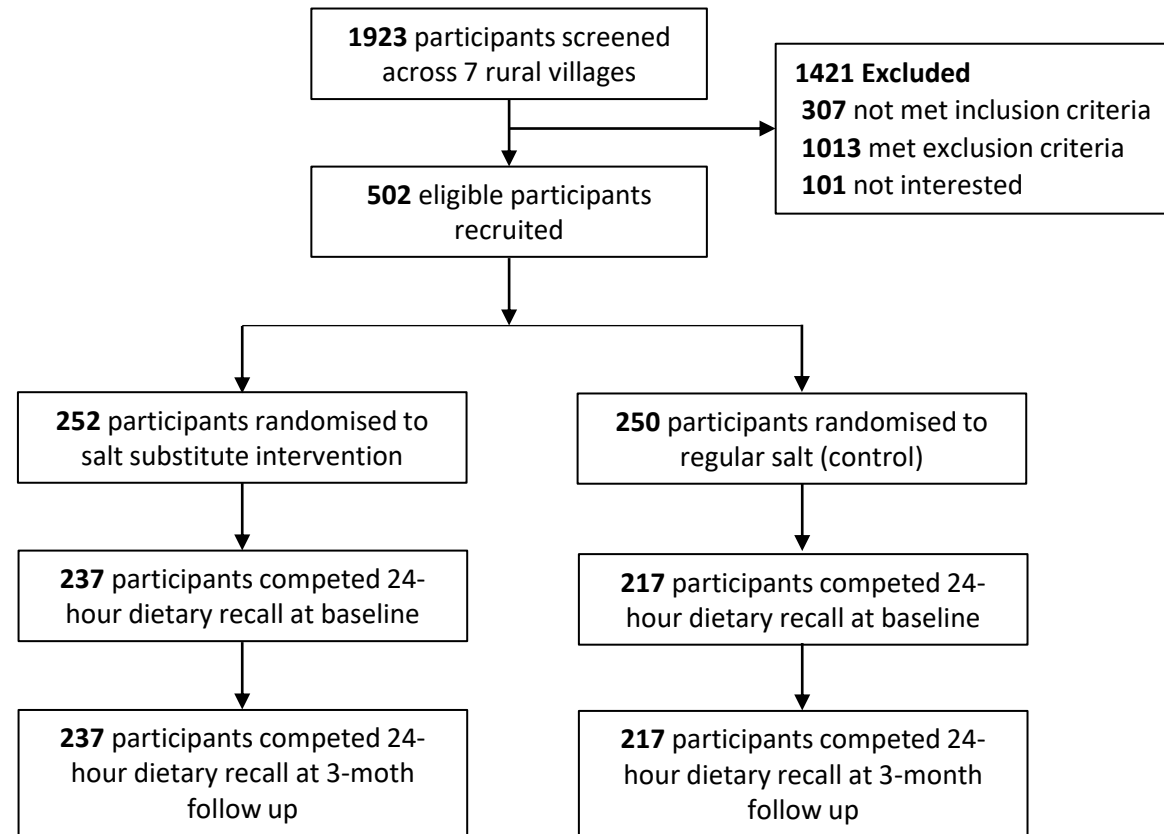

Supplement: Supplementary file 1 — Supplementary material 1. Participant flow diagram [file mmc1.pdf]
