# Supplementary material for: Dietary intakes of hypertensive patients in rural India: Secondary outcomes of a randomised, double-blind, controlled trial
Source: Dialogues Health. 2023 Feb 3;2:100109. doi: 10.1016/j.dialog.2023.100109 (PMC10953904; doi:10.1016/j.dialog.2023.100109)
Supplement: Supplementary file 2 — Supplementary material 2. Criteria for minimum and maximum AHEI scores [file mmc2.pdf]

Supplementary Table 1. Criteria for minimum and maximum AHEI scores

Component	Criteria for minimum score (0)	Criteria for maximum score (10)
Vegetables, servings/d	0	≥5
Fruit, servings/d	0	≥4
Whole grains, g/d		
Female	0	≥75
Male	0	≥90
Sugar-sweetened beverages and fruit juice, servings/d	≥1	0
Nuts and legumes, servings/d	0	≥1
Red/processed meat, servings/d	≥1.5	0
Sodium, mg/d	Highest decile	Lowest decile
Alcohol, drinks/d		
Female	≥2.5	0.5-1.5
Male	≥3.5	0.5-2.0
PUFA, % of energy	≤2	≥10
Total	0	90

Serve of vegetables = 118.30g. All vegetables included except for potatoes and any potato products e.g. French fries.
Serve of fruit = 118.30g for berries and 150g for other fruit. Does not include fruit juice.
Wholegrains = includes brown rice, muesli, porridge, wholegrain breakfast cereals, grain bread, muesli bars.
Sugary drinks = includes sugar-sweetened soft drink, juice, cordials, flavoured milk, flavoured teas and coffees. Excludes any drinks without added sugar. One serve = 226.8g
Nuts and legumes and vegetable protein = includes nuts, peanut butter, soy beans, tofu, baked beans, other beans etc. One serve = 28.35g
Red and processed meat = includes beef, lamb, pork, liver, hamburger buns, meat in pies, devon, hot dogs. One serve of red meat = 113.4g and one serve of processed meat = 42.5g
Alcohol = One serve of alcohol equates to 113.4ml of wine, 340.2ml of beer and 42.5ml of spirits.

Supplementary Table 2. Change in diet quality over the course of the trial according to the AHEI-2010

	Baseline	End of trial	Mean change within group (95% CI)	Baseline	End of trial	Mean change within group (95% CI)	Mean diff between groups at end of trial (95% CI)	P-value for mean diff
**AHEI components, mean (SD)**								
Vegetable score	1.0 (0.1)	1.1 (0.1)	0.1 (-0.1 to 0.3)	1.1 (0.1)	1.4 (0.1)	0.3 (0.2 to 0.5)	0.2 (-0.0 to 0.5)	0.066
Fruit score	0.0 (0.0)	0.0 (0.0)	0.0 (-0.0 to 0.0)	0.0 (0.0)	0.0 (0.0)	-0.0 (-0.0 to 0.0)	-0.0 (-0.1 to 0.0)	0.083
Whole grains score	0.9 (0.1)	0.6 (0.1)	-0.2 (-0.6 to 0.1)	0.9 (0.1)	0.4 (0.1)	-0.5 (-0.8 to -0.1)	-0.2 (-0.7 to 0.3)	0.389
Sugary drinks score	10.0 (0.0)	10.0 (0.0)	0.0 (-0.0 to 0.0)	10.0 (0.0)	10.0 (0.0)	0.0 (0.0 to 0.0)	0.0 (-0.0 to 0.0)	0.175
Nuts and legumes score	5.3 (0.3)	4.7 (0.3)	-0.6 (-1.4 to 0.1)	4.8 (0.3)	4.7 (0.3)	-0.1 (-0.9 to 0.6)	0.5 (-0.6 to 1.6)	0.354
Red meat score	9.5 (0.1)	9.2 (0.1)	-0.3 (-0.7 to 0.0)	9.5 (0.1)	9.2 (0.1)	-0.3 (-0.6 to 0.1)	0.0 (-0.4 to 0.5)	0.843
Sodium score	5.6 (0.2)	5.4 (0.2)	-0.1 (-0.6 to 0.3)	5.8 (0.2)	5.2 (0.2)	-0.6 (-1.0 to -0.1)	-0.4 (-1.0 to 0.2)	0.206
Alcohol score	0.0 (0.1)	0.1 (0.1)	0.1 (-0.1 to 0.2)	0.1 (0.1)	0.1 (0.1)	0.0 (-0.1 to 0.2)	-0.0 (-0.3 to 0.2)	0.631
PUFA score	0.7 (0.1)	0.7 (0.1)	0.1 (-0.1 to 0.3)	0.6 (0.1)	1.2 (0.1)	0.6 (0.4 to 0.8)	0.6 (0.3 to 0.8)	<0.001

Supplementary Table 3. Sodium intake (mg/day) and percent contribution across food categories at baseline, mean (SD)

**Food categories**	**Regular salt (*n* = 217)**		**Salt substitute (*n* = 237)**		**Total (*n* = 454)**
	**Sodium intake (mg/d)**	**% contribution to total sodium**	**Sodium intake (mg/d)**	**% contribution to total sodium**	**Sodium intake (mg/d)**
Beverage (alcoholic)	0 (0)	0 (0)	0 (0)	0 (0)	0 (0)
Beverage (non-alcoholic)	0 (0)	0 (0)	0 (0)	0 (0)	0 (0)
Bread and bakery products	0 (0)	0 (0)	0 (1)	0 (0)	0 (1)
Cereal, grains and products	14 (7)	1 (0)	15 (8)	1 (1)	14 (7)
Dairy and dairy products	36 (35)	2 (3)	58 (53)	3 (4)	47 (47)
Fats and edible oils	0 (0)	0 (0)	0 (0)	0 (0)	0 (0)
Fish and seafood	0 (4)	0 (0)	0 (0)	0 (0)	0 (2)
Fruits and vegetables	98 (65)	5 (3)	84 (67)	4 (4)	91 (66)
Meat, poultry and eggs	13 (32)	1 (2)	18 (52)	1 (3)	16 (44)
Other condiments	14 (142)	0 (4)	26 (206)	1 (8)	20 (179)
Sugar, honey and related products	1 (0)	0 (0)	1 (1)	0 (0)	1 (1)
Discretionary salt	2142 (1193)	91 (6)	2224 (1195)	90 (10)	2185 (1193)
**Total**	2317 (1212)	100 (0)	2425 (1217)	100 (0)	2373 (1214)

Supplementary Table 4. Potassium consumption (mg/day) and percent potassium contribution across food categories at baseline, mean (SD)

Food categories	Regular salt (*n* = 217)		Salt substitute (*n* = 237)		Total (*n* = 454)
	**Potassium consumption (mg/d)**	**% contribution to total potassium**	**Potassium consumption (mg/d)**	**% contribution to total potassium**	**Potassium consumption (mg/d)**
Beverage (alcoholic)	0 (0)	0 (0)	0 (6)	0 (1)	0 (5)
Beverage (non-alcoholic)	1 (1)	0 (0)	1 (1)	0 (0)	1 (1)
Bread and bakery products	0 (0)	0 (0)	0 (2)	0 (0)	0 (1)
Cereal, grains and products	256 (121)	25 (12)	256 (114)	26 (11)	256 (117)
Dairy and dairy products	108 (71)	12 (9)	135 (100)	14 (12)	122 (88)
Fats and edible oils	0 (0)	0 (0)	0 (0)	0 (0)	0 (0)
Fish and seafood	2 (22)	0 (3)	0 (0)	0 (0)	1 (15)
Fruits and vegetables	765 (688)	57 (20)	658 (534)	53 (20)	709 (614)
Meat, poultry and eggs	53 (130)	5 (13)	58 (166)	5 (13)	56 (150)
Other condiments	1 (7)	0 (1)	1 (11)	0 (2)	1 (9)
Sugar, honey and related products	1 (0)	0 (0)	1 (1)	0 (0)	1 (1)
Added salt	5 (3)	1 (0)	11 (93)	1 (2)	8 (67)
**Total**	1191 (683)	100 (0)	1121 (599)	100 (0)	1154 (641)
